# Supplementary material for: A secondary mechanism of action for triazole antifungals in Aspergillus fumigatus mediated by hmg1
Source: Nat Commun. 2024 Apr 29;15:3642. doi: 10.1038/s41467-024-48029-2 (PMC11059170; doi:10.1038/s41467-024-48029-2)
Supplement: Supplementary file 1 — Supplementary Information [file 41467_2024_48029_MOESM1_ESM.pdf]

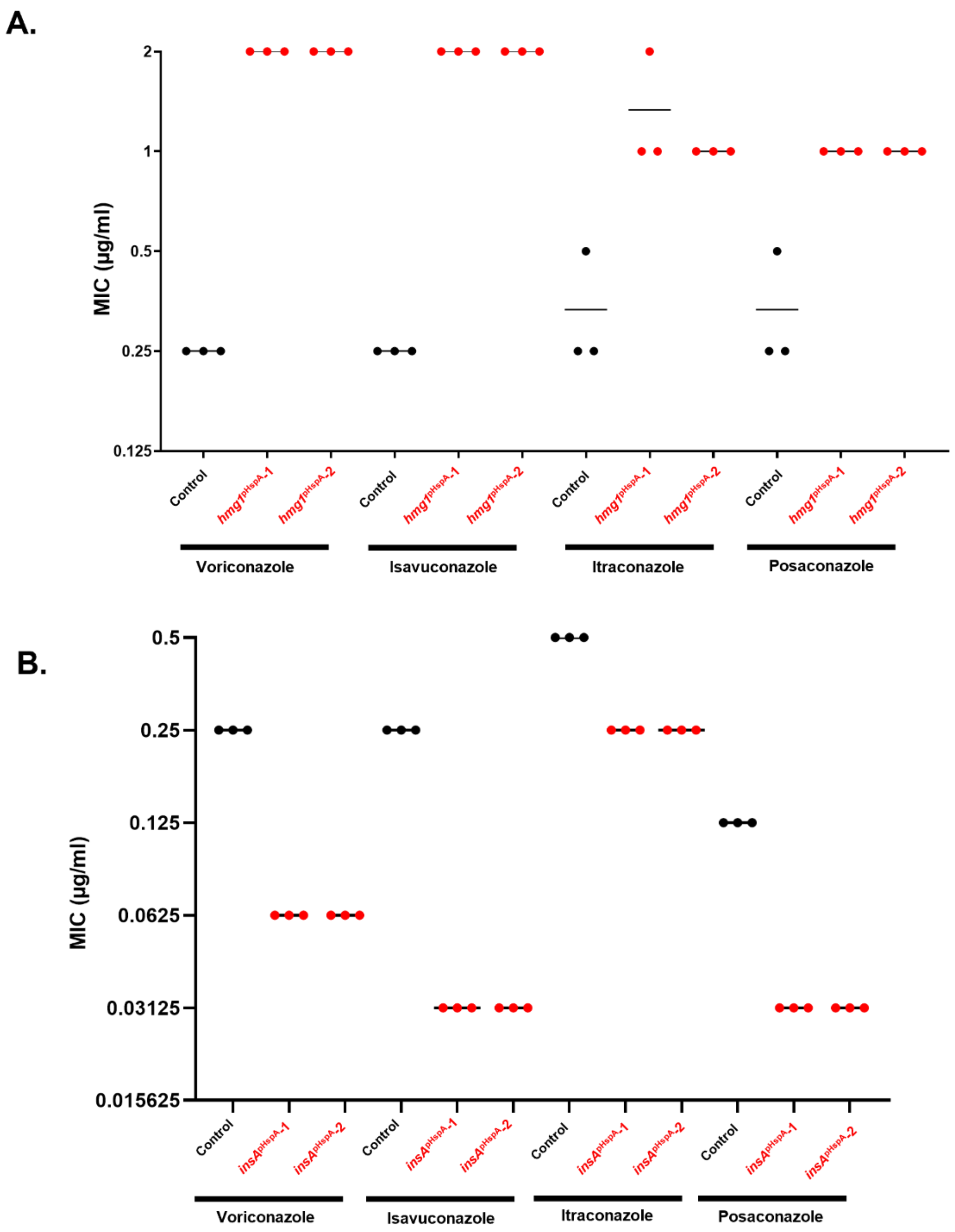

Supplementary Figure 1. A) Susceptibility profiles of the parental control strain (Δ*akuB-pyrG*$^{+}$) versus the *hmg1* overexpression mutants (*hmg1*^{pHspA}-1 and *hmg1*^{pHspA}-2) for voriconazole, isavuconazole, intraconazole, and posaconazole. **B)** Susceptibility profiles of the parental control strain (Δ*akuB-pyrG*$^{+}$) versus the *insA* overexpression mutants (*insA*^{pHspA}-1 and *insA*^{pHspA}-2) for voriconazole, isavuconazole, intraconazole, and posaconazole. CLSI-based minimum inhibitory concentration (MIC) assays were performed in triplicate for each strain. Red font indicates mutant strains with shifts in susceptibility compared to controls. Source data are provided as a Source Data file.

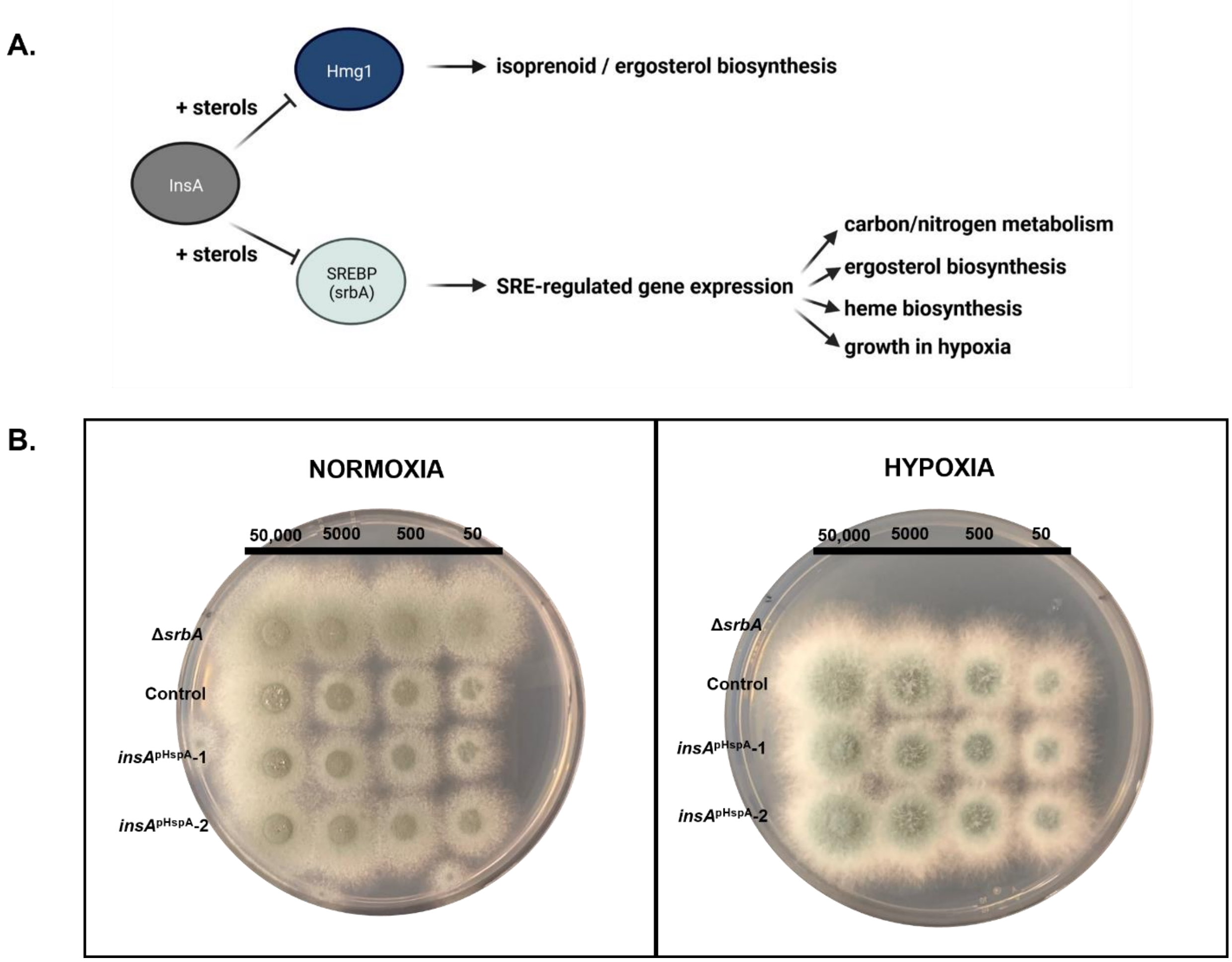

Supplementary Figure 2. A) Schematic of potential sterol-mediated regulatory interactions between InsA and Hmg1 or the sterol regulatory element binding protein (SREBP), SrbA. **B)** Spot dilution assay for the *srbA* deletion strain (Δ*srbA*), the parental control, and the *insA* overexpression mutants (*insA*^{pHspA}-1 and *insA*^{pHspA}-2) in normoxic and hypoxic environmental conditions. Total number of conidia used in each spot inoculum are shown. Source data are provided as a Source Data file.

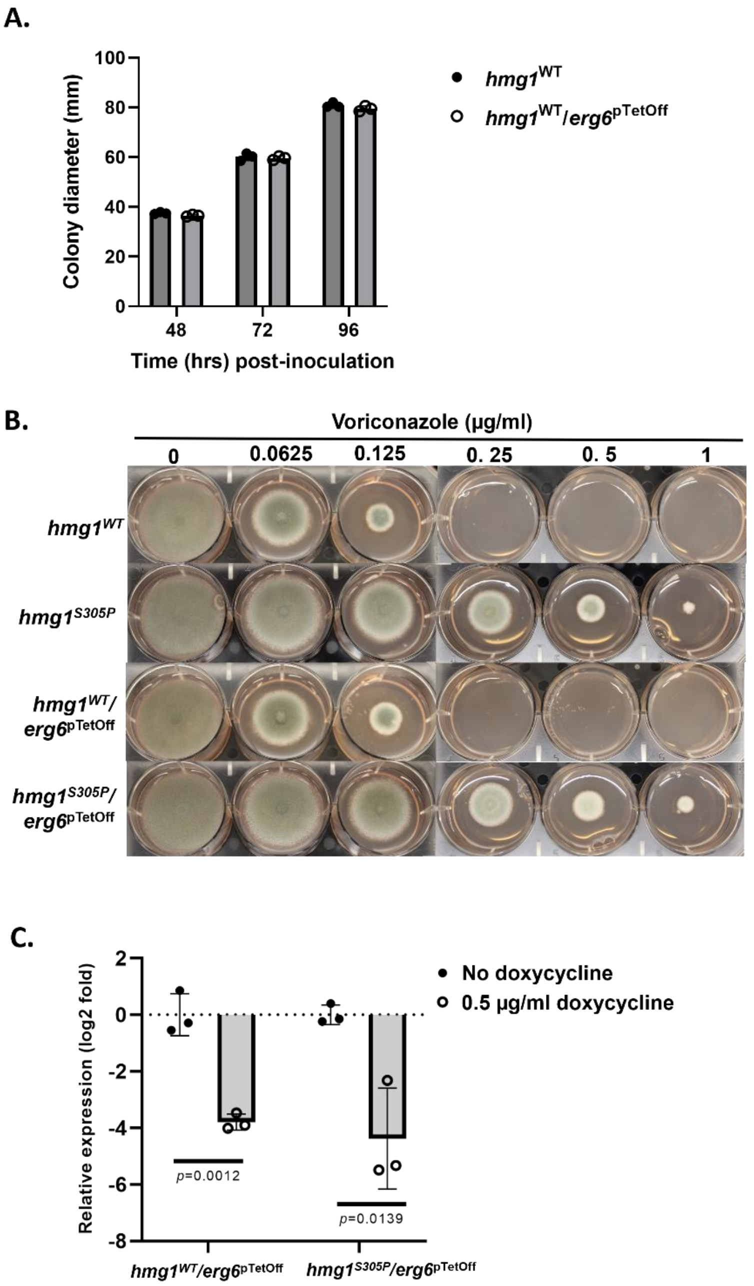

Supplementary Figure 3. Growth and gene expression analyses of the *erg6*pTetOff mutant strains. (A) Colony diameter of the hmg1^{WT} and hmg1^{WT}/*erg6*^{pTetOff} strains at 48, 72, and 96 hrs post-inoculation. Conidia ($1x10^5$) were point-inoculated onto the center of RPMI agar plates and incubated for the indicated times at 37°C. Assays were run in triplicate. **(B)** Voriconazole susceptibility of the indicated strains was assessed as described in Figure 4. **(C)** Doxycycline-mediated *erg6* gene repression was analyzed by RT-qPCR as described in Figure 5. Gene expression was analyzed in triplicate, in the presence and absence of 0.5 µg/ml doxycycline. Data are presented as expression (log_2) in presence of doxycycline relative to no doxycycline conditions. Source data are provided as a Source Data file.

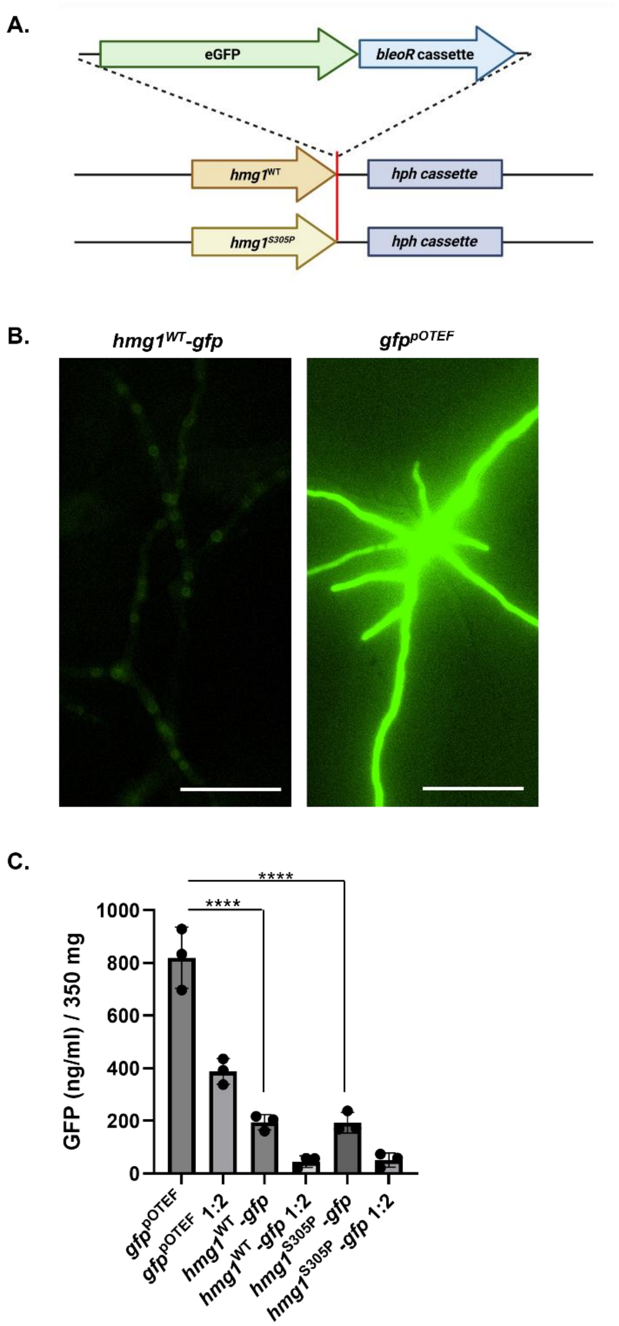

Supplementary Figure 4. Validation of GFP quantitation assay using *A. fumigatus* lysates. A) Schematic for genetic construction of C-terminal GFP fusions of the Hmg1^{WT} and Hmg1^{S305P} proteins. eGFP = enhanced Green Fluorescent Protein; *bleoR* = bleomycin resistance gene; *hph* = hygromycin resistance gene. **B)** Fluorescent micrographs of the *hmg1*^{WT}-*gfp* and *gfp*^{pOtef} strains at 300 ms exposure time. Note the intense fluorescence of the *gfp*^{pOTEF} strain due to high level expression of cytoplasmic GFP. Scale bar = 50 µm. **C)** Quantitation of GFP in lysates of the *gfp*^{pOTEF}, *hmg1*^{WT}-*gfp*, and *hmg1*^{S305P}-*gfp* strains. Samples were tested in biological triplicate under no dilution and 1:2 dilution conditions. Data was analyzed by one-way ANOVA with Tukey's test post-hoc. **** = $p < 0.0001$. Source data are provided as a Source Data file.
