# Supplementary material for: A secondary mechanism of action for triazole antifungals in Aspergillus fumigatus mediated by hmg1
Source: Nat Commun. 2024 Apr 29;15:3642. doi: 10.1038/s41467-024-48029-2 (PMC11059170; doi:10.1038/s41467-024-48029-2)
Supplement: Supplementary file 3 — Description of Additional Supplementary Files [file 41467_2024_48029_MOESM3_ESM.pdf]

Description of Additional Supplementary Files

File Name: Supplementary Data 1
Description: Clinical isolate, oligonucleotide and CRISPR/Cas9 component information. Sheet 1 contains information for each clinical isolate, including isolate name, NBI Bioproject number for genomic data, geographical origin, and antifungal susceptibility profiles to voriconazole (VORI), isavuconazole (ISA), itraconazole (ITRA) and posaconazole (POSA). Sheet 2 lists oligonucleotide primer sequences, as well as protospacer and PAM sequences utilized for mutational analyses.

File Name: Supplementary Data 2
Description: Differentially expressed gene lists of the Hmg1 SSD mutants versus control strains.

File Name: Supplementary Data 3
Description: Sterol profiles of the control and Hmg1 SSD mutant strains in the presence and absence of 0.5 x MIC voriconazole.
