# Supplementary material for: A secondary mechanism of action for triazole antifungals in Aspergillus fumigatus mediated by hmg1
Source: Nat Commun. 2024 Apr 29;15:3642. doi: 10.1038/s41467-024-48029-2 (PMC11059170; doi:10.1038/s41467-024-48029-2)
Supplement: Supplementary file 7 — Reporting Summary [file 41467_2024_48029_MOESM7_ESM.pdf]

nature portfolio

Corresponding author(s): Jarrod R. Fortwendel

Last updated by author(s): Mar 29, 2024

Reporting Summary

Nature Portfolio wishes to improve the reproducibility of the work that we publish. This form provides structure for consistency and transparency in reporting. For further information on Nature Portfolio policies, see our Editorial Policies and the Editorial Policy Checklist.

Statistics

For all statistical analyses, confirm that the following items are present in the figure legend, table legend, main text, or Methods section.

n/a	Confirmed	
☐	☒	The exact sample size (*n*) for each experimental group/condition, given as a discrete number and unit of measurement
☐	☒	A statement on whether measurements were taken from distinct samples or whether the same sample was measured repeatedly
☐	☒	The statistical test(s) used AND whether they are one- or two-sided<br>*Only common tests should be described solely by name; describe more complex techniques in the Methods section.*
☒	☐	A description of all covariates tested
☒	☐	A description of any assumptions or corrections, such as tests of normality and adjustment for multiple comparisons
☐	☒	A full description of the statistical parameters including central tendency (e.g. means) or other basic estimates (e.g. regression coefficient) AND variation (e.g. standard deviation) or associated estimates of uncertainty (e.g. confidence intervals)
☐	☒	For null hypothesis testing, the test statistic (e.g. *F*, *t*, *r*) with confidence intervals, effect sizes, degrees of freedom and *P* value noted<br>*Give P values as exact values whenever suitable.*
☒	☐	For Bayesian analysis, information on the choice of priors and Markov chain Monte Carlo settings
☒	☐	For hierarchical and complex designs, identification of the appropriate level for tests and full reporting of outcomes
☒	☐	Estimates of effect sizes (e.g. Cohen's *d*, Pearson's *r*), indicating how they were calculated

Our web collection on statistics for biologists contains articles on many of the points above.

Software and code

Policy information about availability of computer code

Data collection	Commercially available software used for data collection are described in the Materials and Methods section and included Xcalibur Software for sterol profiling analyses (Thermo Scientific), CFX Maestro for RT-qPCR analyses (bioRad), and Gen5 2.0 for fluorescence measurements (BioTek).
Data analysis	Commercially available software used for data analysis are described in the Materials and Methods section and included Microsoft Excel, SnapGene 4.1.9, GraphPad Prism 7, and GraphPad Prism 10.0.0. For whole-genome sequencing and variant call analyses, all isolate sequences were processed on Terra.bio using the Terra workflow paired-fastq-to-unmapped-bam (https://portal.firecloud.org/?return=terra#methods/gatk/paired-fastq-to-unmapped-bam/10) to run the GATK command FastqToSam. The output unmapped BAM files were then run through the fungal-variant-call-gatk4 workflow (https://github.com/broadinstitute/fungal-wdl/tree/master/gatk4), which implements the GATK HaplotypeCaller (v. 4.1.8.1) for both SNPs and indels. Next, the per-sample GVCF files were combined and genotyped with CombineGVCFs and GenotypeGVCFs. Selected variants were filtered with VariantFiltration and genotypes were filtered with a script in this workflow. The final variant calling format (VCF) file was annotated and given functional predictions using SnpEff (v. 4.3-t), and also filtered for variants with a PASS flag using vcftools (v. 0.1.15). To infer a phylogeny to represent the relationships between isolates, the VCF file was converted into FASTA format using a python script (https://github.com/broadinstitute/broad-fungalgroup/blob/master/scripts/SNPs/vcfSnpsToFasta.py). Maximum likelihood phylogenies were built using RAxML (v. 7.7.8), with the GTRCAT nucleotide substitution model and 1,000 bootstrap replicates. RNAseq and differential gene expression analyses utilized the HiSeq platform (Illumina), HISAT254, and the DE-seq package from Bioconductor.

For manuscripts utilizing custom algorithms or software that are central to the research but not yet described in published literature, software must be made available to editors and reviewers. We strongly encourage code deposition in a community repository (e.g. GitHub). See the Nature Portfolio guidelines for submitting code & software for further information.

Data

Policy information about availability of data

All manuscripts must include a data availability statement. This statement should provide the following information, where applicable:

- Accession codes, unique identifiers, or web links for publicly available datasets
- A description of any restrictions on data availability
- For clinical datasets or third party data, please ensure that the statement adheres to our policy

Whole-genome and transcriptome sequencing data files for the A. fumigatus isolates sequenced as part of this study have been deposited in NCBI SRA under the accession numbers PRJNA985736 and PRJNA991520, respectively. Source data are provided as a Source Data file.

Research involving human participants, their data, or biological material

Policy information about studies with human participants or human data. See also policy information about sex, gender (identity/presentation), and sexual orientation and race, ethnicity and racism.

Reporting on sex and gender	N/A
Reporting on race, ethnicity, or other socially relevant groupings	N/A
Population characteristics	N/A
Recruitment	N/A
Ethics oversight	N/A

Note that full information on the approval of the study protocol must also be provided in the manuscript.

Field-specific reporting

Please select the one below that is the best fit for your research. If you are not sure, read the appropriate sections before making your selection.

☒ Life sciences ☐ Behavioural & social sciences ☐ Ecological, evolutionary & environmental sciences

For a reference copy of the document with all sections, see nature.com/documents/nr-reporting-summary-flat.pdf

Life sciences study design

All studies must disclose on these points even when the disclosure is negative.

Sample size	No statistical tests were utilized to predetermine sample size. As it is the minimum number of replicates required for inferential analysis, at least three biological replicates were utilized for all experiments.
Data exclusions	No data were excluded from analyses
Replication	All experiments were completed with, at least, technical triplicates reading from triplicate biological samples. All replication attempts were successful.
Randomization	Randomization is not relevant to the current study as the statistical comparisons described herein are direct comparisons between strains to analyze the impact of gene mutation (i.e., wild type vs mutant).
Blinding	Blinding was not required for the study as analyses involved direct comparisons between wild type and mutant groups of strains.

Reporting for specific materials, systems and methods

We require information from authors about some types of materials, experimental systems and methods used in many studies. Here, indicate whether each material, system or method listed is relevant to your study. If you are not sure if a list item applies to your research, read the appropriate section before selecting a response.

Materials & experimental systems

n/a	Involved in the study	
☒	☐	Antibodies
☒	☐	Eukaryotic cell lines
☒	☐	Palaeontology and archaeology
☒	☐	Animals and other organisms
☒	☐	Clinical data
☐	☒	Dual use research of concern
☒	☐	Plants

Methods

n/a	Involved in the study	
☒	☐	ChIP-seq
☒	☐	Flow cytometry
☒	☐	MRI-based neuroimaging

Dual use research of concern

Policy information about dual use research of concern

Hazards

Could the accidental, deliberate or reckless misuse of agents or technologies generated in the work, or the application of information presented in the manuscript, pose a threat to:

No	Yes	
☒	☐	Public health
☒	☐	National security
☒	☐	Crops and/or livestock
☒	☐	Ecosystems
☒	☐	Any other significant area

Experiments of concern

Does the work involve any of these experiments of concern:

No	Yes	
☒	☐	Demonstrate how to render a vaccine ineffective
☐	☒	Confer resistance to therapeutically useful antibiotics or antiviral agents
☒	☐	Enhance the virulence of a pathogen or render a nonpathogen virulent
☒	☐	Increase transmissibility of a pathogen
☒	☐	Alter the host range of a pathogen
☒	☐	Enable evasion of diagnostic/detection modalities
☒	☐	Enable the weaponization of a biological agent or toxin
☒	☐	Any other potentially harmful combination of experiments and agents

Precautions and benefits

Biosecurity precautions	All work for this project was conducted in an academic research facility with controlled access that is only available to the laboratory employees and University administrators. All work with the fungal organism was conducted under BSL2 conditions with proper engineering controls for containment.
Biosecurity oversight	All work was conducted under the approval of the University of Tennessee Health Science Center Institutional Biosafety Committee, protocol 22-1019.
Benefits	The work described herein describes a new mechanism by which triazole antifungal drugs inhibit fungal growth. This information can be utilized to understand how pathogenic fungi develop antifungal drug resistance and highlights ways in which currently avaiable antifungals can be improved.
Communication benefits	There is little-to-no risk that the work described here can be utilized to present a public health or national security risk. Aspergillus fumigatus causes invasive disease only in severely immunocomprmised individuals and is not a risk for crops, livestock or ecosystems. Therefore, the benefits of communicating novel mechanisms of drug resistance and antifungal drug activity against this pathogen far outweigh any risk.

Plants

Seed stocks	*Report on the source of all seed stocks or other plant material used. If applicable, state the seed stock centre and catalogue number. If plant specimens were collected from the field, describe the collection location, date and sampling procedures.*
Novel plant genotypes	*Describe the methods by which all novel plant genotypes were produced. This includes those generated by transgenic approaches, gene editing, chemical/radiation-based mutagenesis and hybridization. For transgenic lines, describe the transformation method, the number of independent lines analyzed and the generation upon which experiments were performed. For gene-edited lines, describe the editor used, the endogenous sequence targeted for editing, the targeting guide RNA sequence (if applicable) and how the editor was applied.*
Authentication	*Describe any authentication procedures for each seed stock used or novel genotype generated. Describe any experiments used to assess the effect of a mutation and, where applicable, how potential secondary effects (e.g. second site T-DNA insertions, mosiacism, off-target gene editing) were examined.*
